# Medical News and Miscellany

**Published:** 1897-07

**Authors:** 


### Medical News and Miscellany.

**Dr. Benton** has located at Sabinal, Texas.

---

**Dr. S. M. Applewhite** has located at Uvalde.

---

**Dr. J. E. Hunter,** late of Georgetown, has located at Altoga, Texas.

---

**Dr. S. N. Parks** has located at De Soto, Dallas County, Texas.

---

**Dr. S. S. Southerland** has removed from Netches to Trenton, Tex.

---

**The address** of Dr. J. A. Graves is changed from Pleasant Point to Venus, Texas.

---

**Dr. W. H. Cummings,** a brother to the late Dr. J. E. Cummings, has located at Uvalde.

---

**The address** of Dr. Joseph Kemp is changed from Thorp Springs to Walnut Springs, Texas.

---

**Dr. E. E. Scott,** who has been attending the Kentucky School of Medicine, Louisville, Ky., has returned to Deming's Bridge, Texas.

**Dr. J. T. Harrington** has removed from Abilene to Waco. Dr. J. Cecil Legare, late of Donaldsonville, Louisiana, succeeds Dr. Harrington at Abilene.

---

**Practice For Sale**—With property, furniture. No competition. Good opening for drug store. Price $400. Dr. W. H. Paine, Egan, Tex.

---

**Dr. J. Lewis Smith,** for twenty-five years visiting physician of the New York Foundling Hospital, died in New York City, June 9th (ult.), aged 70 years.

---

**Dr. H. W. Moore,** of Slano, died at his home in that city May 30, 1897. Dr. Moore was a graduate of the Missouri Medical College of the class of 1884.

---

**All subscriptions** beginning with July are now due. We have not dunned our subscribers; but the JOURNAL needs money all the same, and any remittances on account will be gratefully acknowledged.

---

**Something New.**—Dr. Kellogg, of the Battle Creek Sanitarium, is using electric light baths in the treatment of nervous debility and other diseases. It is a novel method of applying radiant heat, and is said to be very beneficial.

---

**Hymeneal.**—Married at Austin, Texas, June 18th (ult.), Mr. Wirt Adams Scott, formerly of Mississippi, to Miss Birdie Caspar, daughter of Dr. F. S. Caspar, the distinguished dentist of Austin; late of Raymond, Miss. No cards; no cake.

---

**Dr. Lusk is Dead.**—Dr. Wm. Thompson Lusk died in New York City, June 12th (ult.), in the 59th year of his age. He died suddenly, of apoplexy. Every one who has ever read his book, "*The Science and Art of Midwifery*," will deplore the too early death of this great author.

---

**Hunt County Medical Association.**—The next quarterly meeting of the Hunt County Medical Association will be held in Commerce, Thursday, July 15, 1897. The session will begin at 10:30 a. m. The JOURNAL is in receipt of a programme, which, however, came too late for publication.

**A New Publication.**—The *X-Ray Journal*, Vol. I., No. 1, May, 1897, has appeared. The purpose of this new monthly is "to give a faithful resume of all X-ray work done in any portion of the globe." The subscription price is $1 per year; the editor, Dr. Heber Robarts, St. Louis, Mo.

---

**Dr. Geo. Sternberg,** Surgeon-General U. S. Army, was elected President of the American Medical Association at the recent meeting in Philadelphia; Dr. J. M. Matthews, of Louisville, Vice-President—the Old Reliable Atkinson, re-elected Secretary, and Denver was chosen as the next place of meeting.

---

**The Journal** learns with deep regret of the death of Mrs. Perkins, wife of our esteemed friend, Dr. A. N. Perkins, the veteran Quarantine Officer of Sabine Pass, and mother of Dr. W. P. Perkins, of Bastrop, La. She died of pneumonia at their home at Sabine Pass, June 4th, ult. We tender our sincere condolence to the bereaved family.

---

**Sex in Societies** (not sects.)—Dr. McLaughlin, of the *Texas Medical News*, calls the State Medical Association "she." How does he know it is a "She?" And worse and worse, Dr. Red, of the *Southwest Texas Medical and Surgical Reporter*, compares the State Medical Association to "a dog that's down." Does not say, tho', whether it is a she dog or a he dog.

---

**A Texas student** takes an honor. James Herschell Thomas, of Texas, was awarded the Professor Glenn Medal by the Vanderbilt University Medical College at the close of the session in March last. The Vanderbilt has its announcement in this issue. Read it, and send to Prof. Douglas, the Dean, for catalogue.

In the twelve years, from 1885 to 1897, inclusive, Vanderbilt Medical College has graduated 2017 medical students.

---

**Mississippi Valley Medical Association.**—The next meeting of the Mississippi Valley Medical Association will be held in Louisville on October 5, 6, 7 and 8, 1897. The president, Dr. Thos. Hunt Stucky, and the chairman of the committee of arrangements, Dr. H. Horace Grant, promise that the meeting will be the most successful in the history of the Association, and this promise is warranted by the well known hospitality of Louisville and Kentucky doctors. Titles of papers should be sent to the secretary, Dr. H. W. Loeb, 3559 Olive street, St. Louis. All railroads will offer reduced rates.

---

**Important Arrest.**—The JOURNAL is in receipt of a letter from an esteemed correspondent in Fannin county, advising that "the notorious Orin Robertson of Texas Health College (the Mound City myth) fame (or in-fame) was arrested in Trenton on the 23rd of June" (ult.). He was arrested on board of a freight train, by City Marshall Bruce Lane, who guarded him until 2 p. m., when the sheriff of Hunt county arrived and took him to Greenville, where he is "wanted" for infanticide.

---

**Prevention of the Rusting of Instruments.**—After an experimental investigation as to the rusting of instruments, it has been found that the process is due to carbonic acid contained in water, and that it is not absolutely prevented by the addition of carbonate of soda, as recommended by some. It was, however, found that rusting can be greatly lessened by first boiling the water before placing instruments in it, since thus the greater part of the carbonic acid is expelled. The most efficient means is to add to the boiled water 0.25 per cent. of sodium hydrate, pure, containing no sulphur. During an operation the instrument should lie in the solution thus prepared. Sharp knives placed in this preparation do not lose their edge in the faintest degree.—*American Practitioner and News.*

---

**Death of Dr. J. E. Cummings.**—The JOURNAL announces, with regret, the death of Dr. Jesse E. Cummings, of Uvalde, Texas. He died on the 30th of April, 1897, after a lingering illness. Dr. Cummings was a native Texan. He was born in 1855, and was in his forty-second year. He was a graduate of the Missouri Medical College. Immediately on graduating he settled in Uvalde for the practice of his profession. He removed to Alpine where he resided for a brief period and went thence to Arizona. A few years ago he returned to Uvalde and continued to reside there till the date of his decease. In 1890 Dr. Cummings was elected county judge, which position he filled for two years. After an interval of two years, he was again elected to that office. As a physician and gentleman he stood high in the community, and his early death is universally deplored;—especially was he dear to the poor, to whom he ministered with untiring zeal night or day, refusing no call, pay or no pay. Dr. Cummings was a brother of our Dr. Josephus Cummings, whose untimely death the JOURNAL was called upon to chronicle only a year ago.

---

**The Austin District Medical Society** held its regular quarterly meeting in Austin on the 24th of June, ult. There was a fair attendance only, but some instructive papers were read, most of which we hope, in time, to give to our readers. We are unable to give a report of the meeting, as we were prevented from being present, and the "able secretary," our own junior—Hudson—has not found time to furnish the JOURNAL with any report. The Association numbers over one hundred, and its interest is unabated.

---

**Chronic Cocainism from Catarrh Snuff.**—A woman was received lately at a Montreal hospital with the trembling hands, staggering gait, insomania, dyspepsia, loss of appetite, etc., of alcoholism, also visual hallucinations, dilatation of the pupils, mental dullness and pronounced moral depravity. She had always been a person of quiet, modest tastes, and her husband asserted that she never took liquor. Asked whether she took any drug, he went home to investigate, and returned with a bottle of Agnew's catarrh powder, a patent remedy which she had been using as a snuff for four or five months, consuming three bottles a week. The bottle held 80 grains and contained 1.75 per cent. cocaine. The therapeutic dose is ¼ to 1 grain.—*Un. Med. du Canada*, April.

---

**A "Windfall."**—Our esteemed contemporary, Bennett, of the Texas Medical Wind-mill, otherwise, *Texas Medical News*, ambitious to be strictly in it with the kids—apparently forgetting his avoirdupoise and his years—mounted a fiery, untamed, bucking-broncho of a bicycle, and hired a dark-skin citizen, in the stilly night; to teach his ponderous pedals to paddle the *pedals*. The darky said the doctor was too fast a man for him; he couldn't go his gait; so, on the brink of a steep slope, in the suburbs of the city, the darky "let go all holds," and the doctor and the cycle went spinning merrily and rapidly down, down, *facilis descendens*, the doctor said, till meeting *something* that objected to such a downward course on the part of so upright a doctor, this *something* headed them off and gave the doctor a "header"-

off. The doctor dislocated his ankle; but quickly set it, on the spot, and was not seriously, but rather painfully, hurt. Fortunately he carried an accident policy, and we are pleased to learn he has been paid well for his mishap, and he paid well for his experience. N. B.—The doctor has gone back to his coupe, and has a bicycle for sale.

---

**Resection of the Liver to Remove a Multilocular Hydatic Cyst.**—This is the first case of the kind on record in which the abdominal cavity was closed after the tumor was removed. The patient had complained of a dull pain in the hepatic region and a tumor was palpated, moving with the liver in respiration. Through an incision parallel with the right costal arch, P. Bruns resected the tumor, the size of a man's fist, after liberating the adherent gall bladder. The hemorrhage was slight, requiring only the ligature of two arteries. The lips of the wound in the hepatic tissue were united with a deep suture of strong catgut and a superficial suture with fine catgut. The abdominal wound was sutured by layers. The wound healed rapidly and the patient left the hospital in three weeks.—From *Beitraege z. Klin. Chir.*, xvii, 1, in the *Sem. Med.*, January 27.

---

**Still Another.**—Under the stimulus of the JOURNAL's constant and earnest advocacy, the profession is being organized in many localities. Attention has been called heretofore to the organization of several societies which are full of promise,—the Brazos Valley, the Northeast Texas, the South Texas, etc., and now comes a cheering report from our subscriber, Dr. U. E. G. Dyer, of Star, Secretary-elect of the society—of the organization of the Mills County Medical Association. It was organized at Goldthwaite, Tex., June 15th, with a membership of fourteen "active, energetic and enthusiastic physicians," the doctor writes. Dr. J. B. Sharp, of Goldthwaite, was elected President, and Dr. A. W. Barton, of same place, Vice-President; Dr. Dyer, Secretary and Treasurer. The JOURNAL extends the glad hand, and bids the new organization godspeed. The good work thus goes merrily on. There is no use in talking; there is only one way to succeed in getting medical legislation against quackery, and that is to thoroughly organize the profession in every village, hamlet and cross road, town, city and county, and send delegates once a year to a big, rousing meeting of the State Association; to unify the profession and let them demand legislation. The demand, coming from a united profession, will be heeded. Our State Association has so run down at the heels that it is difficult to get together one hundred members. We must exert every effort to rebuild it, and the JOURNAL will not let up until we can have meetings of five hundred as of yore. Most of these societies, when organized, make the "Red Back" their official organ, in recognition of its services.

---

**There is no State Board of Health** in Texas. In lieu thereof the public health department, consisting of a system of coast and border quarantine, and general supervision of internal sanitation 's administered by a State Health Officer. In each county there is a county physician, appointed by the county judge—subordinate to the State Health Officer only in matters relating to infectious or contagious disease. This statement is made for the information of the public, as numerous letters are received by the State Health Officer addressed to the State Board of Health, or the Secretary of the Board—asking information, usually about the law regulating the practice—a matter with which the State Health Officer has no connection. Most of these letters contain no stamp for reply. Whenever the writer is considerate enough to enclose return postage, the information is cheerfully given, although it is a matter entirely foreign to the duties of the State Health Officer.

---

**Army Positions.**—In view of the crowded condition of the ranks of the medical profession,—and the constant additions every year being made to it by the scores of schools; in view of the fact that there is not,—cannot be a living practice for all the graduates of medicine, and the struggle that nesessarily ensues for "locations," it is, indeed, remarkable that there are not more applications for appointment in the medical corps of tee United States army and navy. So few are the applicants that Uncle Sam has, really, to advertise for them. It would seem to us that our bright young graduates wouldn't want a better thing than to get to be an army or navy surgeon. Of course, the examination is strict; it must be, but that should be a stimulus to one's ambition, rather than an impediment. Dr. Dawbarn, whose advertisement see, in this issue, makes a special business of coaching young men for these examinations. A

letter to him will bring information that will interest those who would like to wear the chevrons of an army or navy medical staff officer. Write to him.

---

**A Wine of Iron and Quassia.**—The *Gazette hebdomadaire de medecine et de chirurgie* for May 20th, gives the following formula:

| | | |
|---|---|---|
| ℞ | Tincture of quassi .......................... | 30 parts. |
| | Pyrophosphate of iron and sodium...... | 5 parts. |
| | Malaga wine .................................. | 1,000 parts. |

M. S.: A tablespoonful before each of the two principal meals.—*New York Medical Journal.*

---

**Lesions of the Spinal Cord in Cases of Amputations of the Fingers.**—The necropsy of a recent case has strikingly confirmed the modern assertion that the section of a nerve determines lesions at a distance, in the nerve's originating center. In this case, described and illustrated in the *Presse Med.* of June 2, the lesions in the spinal cord corresponded in every particular and almost exclusively to the innervation of the parts amputated. An interesting feature of the case was that the amputation was congenital. The woman was 60, and a cancer was located in the cervix uteri.—*Journal Am. Med. Ass'n.*

---

**Section Officers of the State Medical Association.**—

*Surgery.*—Chairman, B. E. Hadra, San Antonio; Secretary, J. R. Stuart, Houston.

*Practice.*—Chairman, A. B. Gardner, Bellville; Secretary, J. S. Wooten, Austin.

*Obstetrics.*—Chairman, C. L. Gwyn, Galveston; Secretary, T. L. Kennedy, Galveston.

*Gynecology.*—Chairman, H. K. Leake, Dallas; Secretary, S. F. King, Sherman.

*Pediatrics.*—Chairman, T. J. Bennett, Austin; Secretary, E. A. Woldert, Tyler

*Forensic Medicine and Hygiene.*—Chairman, F. S. White, Terrell; Secretary, S. E. Hudson, Austin.

*Ophthalmology.*—Chairman, R. F. Miller, Sherman; Secretary, S. L. Terrell, Dallas.

*Necrology.*—Chairman, E. D. Capps, Fort Worth.

By appointments of Vice-Presidents S. C. Red, A. C. Scott and C. M. Alexander.

**National Prison Association Congress.**—The city council and the board of trade of Austin, having extended an invitation to the National Prison Association of the United States to hold their next annual prison congress in the city of Austin, the invitation was accepted by the association during their meeting held at Wilwaukee in September, 1896, and the next congress will be held at Austin, October 16th to 20th, 1897.

The members of the association comprise principally wardens and directors of prisons and reformatories, prison chaplains, prison physicians, penologists and philanthropists. The papers read, addresses delivered and discussions to be had during the congress will cover all phases of prison management, reform and prevention of crime and pauperism, and are highly interesting and instructive. The citizens of Austin will provide a place of meeting, obtain favorable railroad rates, extend a welcome to the delegates, and especially to awaken interest in the subject and secure good attendance at the meetings, so that we may derive all the benefit that may accrue to our people from the treatment of the important questions. Gov. Culberson will lend his hearty co-operation to make the congress a grand success.

The citizens of Austin have organized local committees, and will make the meeting a success.

---

**Radiant Heat in the Treatment of Ulcers of the Leg.**—According to the *Lancet* for May 29th, Dr. Colleville, of the Rheims faculty of medicine, treats ulcers of the leg by exposing them to heat. The flame of a Bunsen burner is made to impinge on a square plate of metal that will stand heating, so as to bring it to a dull red heat, and the ulcer is exposed to the action of the heat at a distance of about ten inches, the rest of the limb being protected with bandages. The temperature at the ulcer is about 113° F., which is easily borne, and the flame is so regulated as to maintain this temperature during the whole exposure, from twenty minutes to an hour. The surface is then found to be glazed over, and large granulations are to be seen through the semi-transparent coating. It is thought best to leave the ulcer exposed to the air for some time, and when it is dressed care should be taken that its surface is not touched by the aseptic gauze or other materials used. It is said that some improvement is generally experienced by the patient after the first sitting, and cicatrization is completed in from five to twenty-five applications. In the latter sittings, when the ulcer is nearly healed, a more moderate heat may be employed. If gas is not available, the heat of the sun or of a fire may be used. Dr. Colleville attributes the beneficial effect to the combined action of the heat, light and ventilation.—*New York Medical Journal.*

---

**The New York Medical Leagues Puts the Brake on More Dispensaries.**—We gleam from the press of New York that the New York Medical League has commenced active operations in the matter of medical charities abuses. On April 19th, a committee of the league appeared before the New York State Board of Medical Charity in opposition to the application for a charter for the St. Bartholomew Dispensary, claiming that the institution of a new dispensary in a neighborhood already amply provided for by medical and surgical services, was unnecessary and tending to promote pauperism. The board accepted the views of the league and refused the grant of charter. The Pasteur Institute also applied for a license to open another dispensary, and the league objecting, urged the refusal of recognition by the board. Here, as in the preceding case, the league was sustained by the State board of charities and the charter was not granted. Let the good work on.—*Exchange.*

---

**Our Colleges.**—At this season the young students of medicine, who have been reading at home or in the office of some practitioner of medicine, are beginning to cast around for the best place to matriculate and graduate. It is a matter of great importance; one which cannot be too well considered. It is a matter in which it will not do to make any mistake; for a mistake in the beginning may be embarrassing all one's life. One should select a school of established character and reputation for able teachers and thorough teaching; one whose requirements for matriculation are high, and whose requirements for graduation are difficult; for no one prizes that which is easily won; and a diploma from a school where the examinations are hard to pass, and require knowledge, will always be something to be proud of. Now, we are not going to discriminate by recommending any school. We will just call attention to the claims of the schools that advertise with us, and say to readers "investigate and choose for yourself."

We call attention, especially, to the announcements of the

Tulane University Medical Department, at New Orleans.
Louisville Medical College, always a favorite with Texans.
Texas Medical College at Galveston.
Fort Worth Medical College at Fort Worth.
Old Jefferson Medical College.
Creighton Medical College of *Omaha.*
University of Nashville Medical Department.
University College of Medicine, Richmond, Va.
Chattanooga Medical College.
Central Medical College, St. Joseph, Mo.
University, Tennessee, Medical Department.
Vanderbilt University Medical Department.
Southern Medical College, Atlanta.
Medical Department Columbian University.
Marion Sims Medical College, St. Louis.
Bellevue Hospital Medical College, N. Y.
N. W. University Medical College, Chicago.
University, Pennsylvania, Medical College, Philadelphia.

These are all high class schools, and a student will be safe in selecting either one for his *alma mater.* In writing to the Dean or Secretary, please mention this notice and oblige us.

